# Supplementary figures and images for: Early line and hook fishing at the Epipaleolithic site of Jordan River Dureijat (Northern Israel)
Source: PLoS One. 2021 Oct 6;16(10):e0257710. doi: 10.1371/journal.pone.0257710 (PMC8494375; doi:10.1371/journal.pone.0257710)

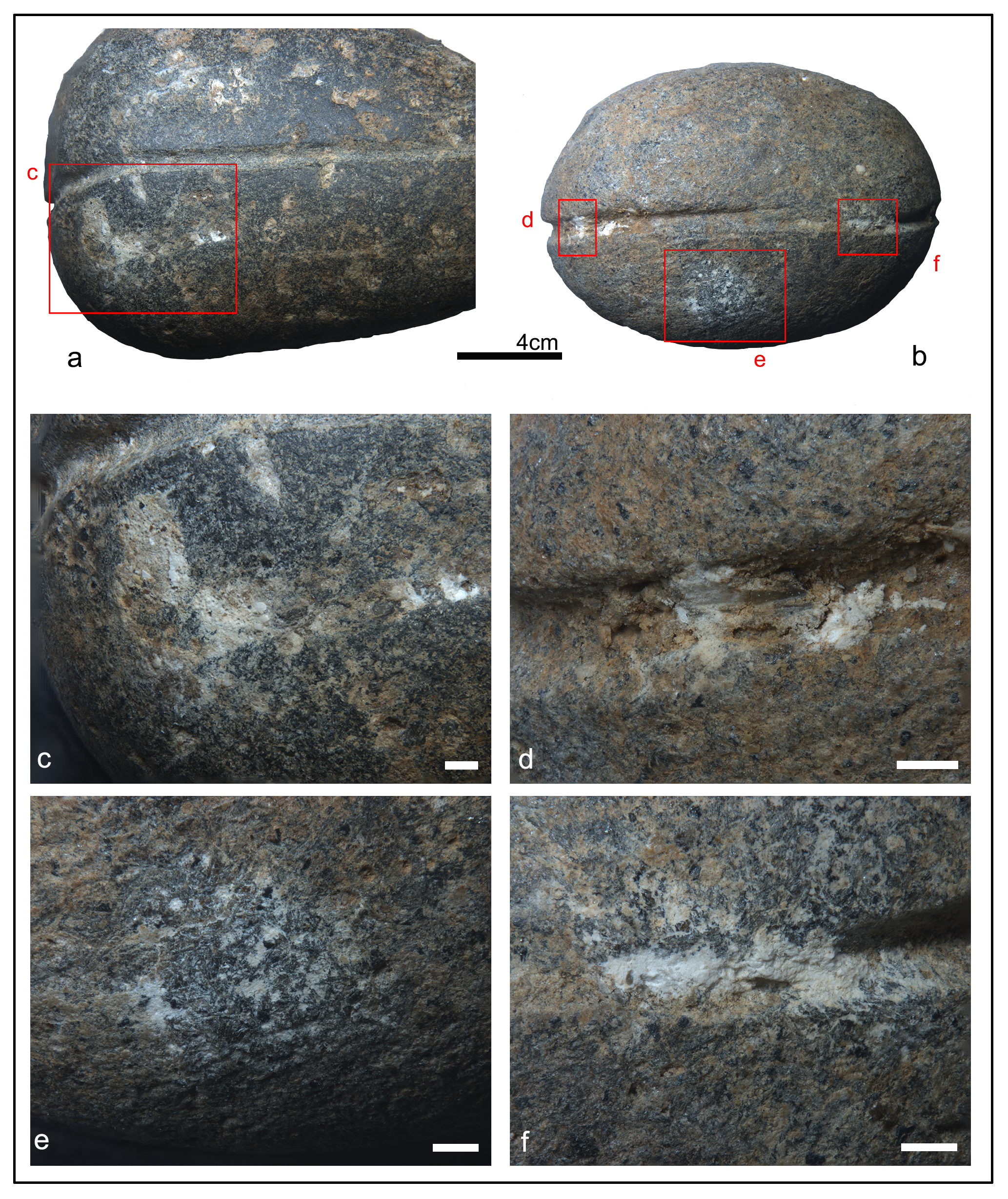

Supplement: S1 Fig — a) #100; b) #103; c) residue extraction n. 1; d) residue extraction n. 2; e) Residue extraction n. 3; f) residue extraction n. 4. scale bars measure 500 μm. (TIF) [file pone.0257710.s001.tif]

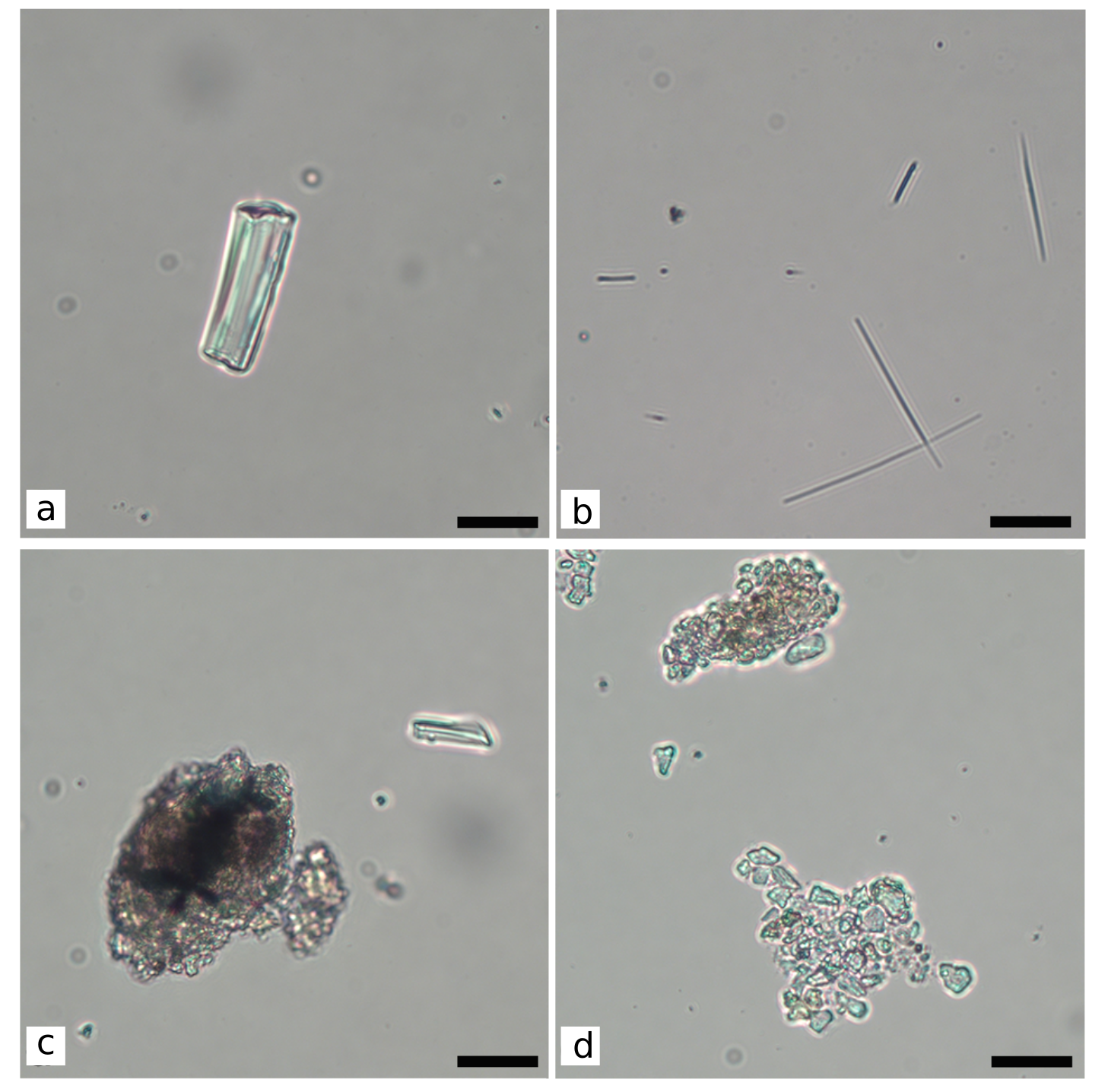

Supplement: S2 Fig — a) Shell fragments; b) raphides; c) sediment particles and a shell fragment; d) sediment particles. Scale bars measure 20 μm. (TIF) [file pone.0257710.s002.tif]
