# Supplementary material for: Early line and hook fishing at the Epipaleolithic site of Jordan River Dureijat (Northern Israel)
Source: PLoS One. 2021 Oct 6;16(10):e0257710. doi: 10.1371/journal.pone.0257710 (PMC8494375; doi:10.1371/journal.pone.0257710)
Supplement: S1 Table — (DOCX) [file pone.0257710.s003.docx]

**Supplementary Table 1: JRD bone fish hook measurements**

|  | Layer | Length (mm) | Width (mm) | Hook size (mm) | Gape (mm) | Bite (mm) | Angle (deg.) | Bend cs height (mm) | Bend cs thickness (mm) | Bend cs size (mm) | Bend cs y/z |
| --- | --- | --- | --- | --- | --- | --- | --- | --- | --- | --- | --- |
| Hook 1 | 3b | 21.2 | 12.5 | 16.3 | 7.8 | 15.7 | -10.3 | 4.0 | 1.4 | 2.4 | 2.8 |
| Hook 2 | 3b | 21.6 | 10.4 | 15.0 | 7.1 | 9.6 | -1.6 | 2.7 | 2.8 | 2.8 | 1.0 |
| Hook 3 | 3b | 13.8 | 7.9 | 10.4 | 4.3 | 8.5 | -7.9 | 2.7 | 1.6 | 2.1 | 1.7 |
| Hook 4 | 3a | 15.6 | 6.3 | 9.9 | 3.3 | 9.5 | -4.1 | 3.4 | 1.7 | 2.4 | 2.0 |
| Hook 5 | 3b |  |  |  |  |  |  | 5.1 | 2.9 | 3.9 | 1.8 |
| Hook 6 | 3a |  |  |  |  |  |  | 3.6 | 1.2 | 2.0 | 3.0 |
| Hook 7 | 3a |  |  |  |  |  |  | 2.2 | 1.6 | 1.9 | 1.4 |
| Hook 8 | 3b |  |  |  |  |  |  | 2.3 | 1.5 | 1.9 | 1.5 |
| Hook 9 | 3a |  |  |  |  |  |  | 2.1 | 0.8 | 1.3 | 2.6 |
| Hook 10 | 4 |  |  |  |  |  |  | 2.2 | 1.4 | 1.7 | 1.6 |
| Hook 11 | 3a | 19.2 | 8.7 | 12.9 | 4.9 | 12.3 | -3.7 | 3.5 | 2.2 | 2.8 | 1.6 |
| Hook 12 | D3 | 15.6 | 10.7 | 12.9 | 5.4 | 11.8 | -19.5 | 3.2 | 3.0 | 3.1 | 1.1 |
| Hook 13 | D8 | 51.3 | 17.4 | 29.8 | 9.9 | 30.6 | -2.0 | 6.5 | 3.6 | 4.9 | 1.8 |
| Hook 14 | 3a |  |  |  |  |  |  | 5.3 | 3.3 | 4.2 | 1.6 |
| Hook 15 | 3-0 |  |  |  |  |  |  | 2.8 | 1.9 | 2.3 | 1.5 |
| Hook 16 | 3-0 |  |  |  |  |  |  | 3.7 | 2.1 | 2.8 | 1.8 |
| Hook 17 | D4 |  |  |  |  |  |  | 2.9 | 1.7 | 2.2 | 1.7 |
| Hook 18 | 3-0 |  |  |  |  |  |  | 3.0 | 2.2 | 2.6 | 1.4 |
