# Supplementary material for: Early line and hook fishing at the Epipaleolithic site of Jordan River Dureijat (Northern Israel)
Source: PLoS One. 2021 Oct 6;16(10):e0257710. doi: 10.1371/journal.pone.0257710 (PMC8494375; doi:10.1371/journal.pone.0257710)
Supplement: Attachment — Submitted filename: Response to Reviewers.docx [file pone.0257710.s004.docx]

Response to Editor and Reviewer's Comments

*Please see our responses to each of the reviewer and editor's comments after each suggestion below (in italics).*

Editor’s Comments:

4. Thank you for stating the following in the Acknowledgments Section of your manuscript:

[The JRD excavation (Israel Antiquity Authority License # G-75/2018) is supported by grants to GS from the following agencies: The Israel Science Foundation (Grant #918/17), the Brennan Foundation (two grants), the Wenner-Gren Foundation, National Geographic Society, Irene Levi Sala Care Archaeological Foundation, Tel Hai College and MIGAL research institute. This research was also supported by grants from the European Research Council(Starting Grant Project HIDDEN FOODS, grant no. 639286)awarded to EC and the National Science Foundation (BCS-1842087) awarded to NM.Analyses at TraCEr (Neuwied, Germany) were supported by the Römisch Germanisches Zentralmuseum –Leibniz Research Institute for Archeology by German Federal and Rhineland Palatinate funding(Sondertatbestand “Spurenlabor”).]

We note that you have provided funding information that is not currently declared in your Funding Statement. However, funding information should not appear in the Acknowledgments section or other areas of your manuscript. We will only publish funding information present in the Funding Statement section of the online submission form.

Please remove any funding-related text from the manuscript and let us know how you would like to update your Funding Statement. Currently, your Funding Statement reads as follows:

 [Research is supported by the Israel Science Foundatio - [www.isf.org.il](http://www.isf.org.il) (Grant #918/17) granted to G. Sharon.
This research was also supported by grants from the European Research Council - <https://erc.europa.eu> (Starting Grant Project HIDDEN FOODS, grant no. 639286) awarded to E. Cristiani and the National Science Foundation - <https://www.nsf.gov> (BCS-1842087) awarded to N. Munro.
The funders had no role in study design, data collection and analysis, decision to publish, or preparation of the manuscript.]

Please include your amended statements within your cover letter; we will change the online submission form on your behalf

*The funding related text was removed from the acknowledgments and the funding statement was updated.*

5. We note that Figure 1 in your submission contain map images which may be copyrighted. All PLOS content is published under the Creative Commons Attribution License (CC BY 4.0), which means that the manuscript, images, and Supporting Information files will be freely available online, and any third party is permitted to access, download, copy, distribute, and use these materials in any way, even commercially, with proper attribution. For these reasons, we cannot publish previously copyrighted maps or satellite images created using proprietary data, such as Google software (Google Maps, Street View, and Earth). For more information, see our copyright guidelines: <http://journals.plos.org/plosone/s/licenses-and-copyright>.

 We require you to either (1) present written permission from the copyright holder to publish these figures specifically under the CC BY 4.0 license, or (2) remove the figures from your submission:

*Figure 1 is comprised of a single aerial picture and 3 maps. All parts of Figure 1 were produced as part of the JRD research project and Gonen Sharon – head of the JRD excavation project, holds the rights to all parts. No use was made in any outside source (such as google earth etc.) in the creation of Figure 1.*

*In addition, please not that we uploaded a new corrected version of Figure 11 with corrected numbering.*

Reviewer's Comments:

Reviewer #1: The article presents very interesting finds of early fishing equipment. Research methods include both archaeological and scientific analyses. Results obtained during this research are quite persuasive. Discussion touches various sides of ancient line fishing in the Jordan River valley and in a broader context. Conclusions are clear and supported by the analyzed data. The article should be published as it is.

*Thank you.*

Reviewer #2: In summary, this is a well written, well researched, comprehensive and data rich paper that should be published with only a few minor changes. I have made some comments about useful changes.

Required change
line 125. Possible straight fish hooks (gorges or hameçon droits) were in use during the Magdalenian when artistic representations of fish appear (34), but the earliest fish hooks known thus far are made of shell and come from the cave of Jerimalai in East Timor. The site of Jerimalai has now been renamed Asitau Kura at the request of the traditional owners. Maybe it should say ' but the earliest fish hooks known thus far are made of shell and come from the cave of Asitau Kuru (formerly known as Jerimalai) in East Timor.'
Shipton, C., S. O'Connor, N. Jankowski, J. O'Connor-Veth, T. Maloney, S. Kealy and C. Boulanger 2019 A new 44,000-year sequence from Asitau Kuru (Jerimalai), Timor-Leste, indicates long-term continuity in human behaviour. Archaeological and Anthropological Sciences 11(10):5717–5741.

*The site name was changed as suggested and the reference was added*

Line 127 Jerimalai should be changed to Asitau Kuru. Please make this change throughout and reference the Shipton et al. paper

*These changes were made and the reference was added.*

Suggestions for useful changes or additions
Line 118. Barbed bone artefacts which are thought to have been used as projectile tips for spears are also found in East Timor in the site of Matja Kuru 2 where they are dated earlier than 30ka. As there are no large mammals on the island of Timor at this time it was argued that they may have been used in a maritime context for spearing large fish or marine animals see

O’Connor, S., G. Robertson and K.P. Aplin 2014 Are osseous artefacts a window to perishable material culture? Implications of an unusually complex bone tool from the Late Pleistocene of East Timor. Journal of Human Evolution 67:108–119.

*This reference was added and the text was updated accordingly.*

It might also be useful for the authors to look at a recent paper on the fish hook assemblage from Makpan Cave in Alor Island which is a large assemblage which also shows all stages of the production sequence as well as the items used for their production such as files.
Michelle C. Langley, Sue O’Connor, Shimona Kealy & Mahirta (2021): Fishhooks, Lures, and Sinkers: Intensive Manufacture of Marine Technology from the Terminal Pleistocene at Makpan Cave, Alor Island, Indonesia, The Journal of Island and Coastal Archaeology, DOI: 10.1080/15564894.2020.1868631

*This paper describes another assemblage of fishhooks, part of which dates to the Late Pleistocene and also argues for the early use of lures. We added the reference and tweaked our text in several places to account for this new data.*

*Thank you and all the best*

*Gonen Sharon*
